# The potential role of diffusion weighted imaging in the diagnosis of early carotid and vertebral artery dissection

**DOI:** 10.1007/s00234-021-02842-4

**Published:** 2021-11-13

**Authors:** Mohammad Almohammad, Mete Dadak, Friedrich Götz, Frank Donnerstag, Anita Blanka Tryc, Nima Mahmoudi, Mike P. Wattjes

**Affiliations:** 1 Department of Diagnostic and Interventional Neuroradiology, Hannover Medical School, Carl-Neuberg-Straße 1, 30625 Hannover, Germany; 2 Department of Radiology, St. Vincenz Hospital Paderborn, Paderborn, Germany; 3 Department of Neurology, Hannover Medical School, Carl-Neuberg-Straße 1, 30625 Hannover, Germany

**Keywords:** Dissection, Diffusion weighted imaging, DWI, Intramural hematoma, Stenosis

## Abstract

**Purpose:**

To investigate the role of the diffusion weighted imaging (DWI) in the acute dissection of internal carotid artery (ICA) and vertebral artery (VA) and assessing the length of intramural hematoma (IMH), caused by dissection.

**Methods:**

We analyzed 28 patients presenting with a dissection of the ICA and/or VA with respect to the presence of high signal intensity areas on DWI suggestive of dissection and 20 control subjects without arterial dissection, some with and some without atherosclerotic lesions. ICA or VA dissection was defined by clinical and imaging, computed tomography angiography (CTA), MR angiography (MRA), and digital subtraction angiography (DSA) findings. The length of DWI hyperintensity was compared to length of the occlusion or stenosis on the angiographic examination.

**Results:**

In 28 patients, 30 dissected arteries were analyzed. Time intervals from the onset of the first clinical symptoms to the radiological evaluation ranged from 1.5 h to 42 days. In 28 (93%) of the dissections, a high signal intensity of the affected artery was present on DWI. The measurement of the dissection length on DWI compared to DSA showed a mean deviation of 2.7 mm and a standard deviation of 3.7 mm.

**Conclusion:**

DWI is a highly sensitive and valuable pulse sequence for the detection of dissected cervical arteries even in the first hours after symptom onset. In contrast to CTA and MRA, DWI can be a potential tool for a reliable measurement of the dissection length.

## Introduction

Arterial dissection (AD) is characterized by an intima tear leading to an intramural hematoma and a subsequent splitting of vessel wall layers. This causes a stenosis or an occlusion and potentially an aneurysmal dilatation of the vessel [1]. The annual incidence of internal carotid artery (ICA) dissections is 1.72 per 100,000, and the annual incidence rate of vertebral artery (VA) dissections is 0.97 per 100,000 [2]. Clinically, cervical (carotid or vertebral) artery dissection (CAD) frequently presents with unilateral head and neck pain and may accompanied by a partial Horner’s syndrome (oculosympathetic palsy) and followed in the next hours or days by cerebral and/or retinal ischemia. This classic clinical triad is present in less than one third of patients. However, if the patient presents with two of these three symptoms, this is highly suggestive of a CAD [1]. In young adults, CAD is a frequent cause of ischemic stroke representing about 10–20% [3–5]. In addition to the characteristic clinical presentation, the diagnosis of CAD is based on imaging, either ultrasound, computed tomography angiography (CTA), magnetic resonance angiography (MRA), or digital subtraction angiography (DSA). Magnetic resonance imaging (MRI) is capable of detecting intramural hematoma (IMH) on T2-weighted and fat suppressed T1-weighted images.

Although the diagnostic strategy regarding CAD is quite straightforward, an early and accurate diagnosis can be challenging, particularly in young patients in whom CAD is one of the most common missed diagnosis of ischemic stroke [6]. In the acute stage (24–48 h after symptom onset), IMH of the dissected artery may not be detected on both T2- and T1-weigthed images potentially leading to delayed or even missed diagnosis of CAD [7]. It has been suggested that the IMH may show isointense signal intensities on fat saturated T1-weighted images compared to the adjacent surrounding tissues up to 7 days after symptom onset challenging an early accurate diagnosis [8]. Therefore, there is a so far unmet medical need for additional imaging markers supporting the clinical diagnosis of CAD. Few studies have suggested that diffusion weighted MRI (DWI) may have potential added value in the early detection of IMH and CAD. A case series of four patients and an additional single case study demonstrated that dissected vertebral arteries can show a high signal intensity on DWI [9, 10]. Another study suggested that the DWI might be a useful complement to conventional MRI for identifying hemorrhage of carotid plaques and differentiation between IMH and intraplaque hemorrhage (IPH) [11]. However, larger confirmatory studies are lacking.

The aim of our study was to investigate the role of the diffusion weighted imaging (DWI) in detecting the acute dissection of ICA and VA. The second aim was and to assess the length of the dissected area.

## Materials and methods

This was a retrospective study including patients with confirmed CAD based on clinical and radiological characteristics. This study has been approved by our local institutional review board, and written informed consent was obtained from all participants regarding the use of clinical, paraclinical, and imaging data for research purposes.

### Inclusion criteria

The inclusion criteria were a confirmed diagnosis of an arterial dissection in one or more of the VA or ICA based on clinical presentation and radiological findings. Clinical presentation is as follows: typical signs and symptoms of the cervical arterial dissection (like headaches, neck pain, Horner syndrome) as well as typical stroke-like symptoms (e.g., hemiparesis, hemihypesthesia, ataxia, aphasia, dysarthria, neglect, impaired vision). The time point of the first neurological symptom suggestive of dissection has been considered as CAD onset. We did not differentiate between trauma related or spontaneous CAD. Patients had to meet the following radiological criteria: The dissection had to be confirmed by at least one of the following examinations: MRI (T1w, T2w, and/or MRA), CTA, and/or DSA. In addition, the availability of brain/head and neck MRI including a DWI focusing on the area of the affected artery was mandatory. The final inclusion criterion was the availability of at least one of the angiographic imaging modalities (MRA, CTA or DSA) focusing on the affected artery in order to assess the length of dissected area.

### Patient selection

From our continuously maintained stroke database, we screened 48 patients presenting with clinical symptoms suggestive of CAD between January 2014 and December 2018. Eighteen patients were excluded because of missing imaging data (no MRI and/or no DWI available). Two further patients were excluded, one patient because of absence of medical history suggestive of dissection and typical dissection signs and symptoms and the other one because of absence of the imaging-based confirmation of the dissection.

To evaluate the specificity, we created a control group and screened for patients without CAD (with or without atherosclerotic lesions) and patients with a stenosis or occlusion of either of the ICA or the VA.

### MR imaging acquisition

Image acquisition was performed on whole-body MR systems operating at 1.5 Tesla (AERA, AVANTO Siemens, Erlangen Germany) or 3 Tesla (VERIO, SKYRA, Siemens, Erlangen Germany). The acquisition protocol was performed in the setting of acute stroke and included in all patients a scanner optimized axial fluid-attenuated inversion recovery (FLAIR) (TR 7000–9000 ms, TE 81–83 ms, ST 5.5 mm), axial and coronal DWI (TR 7000–7600 ms, TE 91 ms, ST 5.5 mm), and time of flight (TOF) MR angiography. The routine *b*-value in the DWI was 0 and 1000 s/mm^2^ (the DWI with the *b*-value of 1000 was used to assess the dissected area), and the routine slice thickness was 5.5 mm. The axial and coronal sections of DWI were used mainly to detect cerebral infarction. We used these DWI sequences to detect CAD and to measure its length.

### Image analysis

**Fig. 1:**
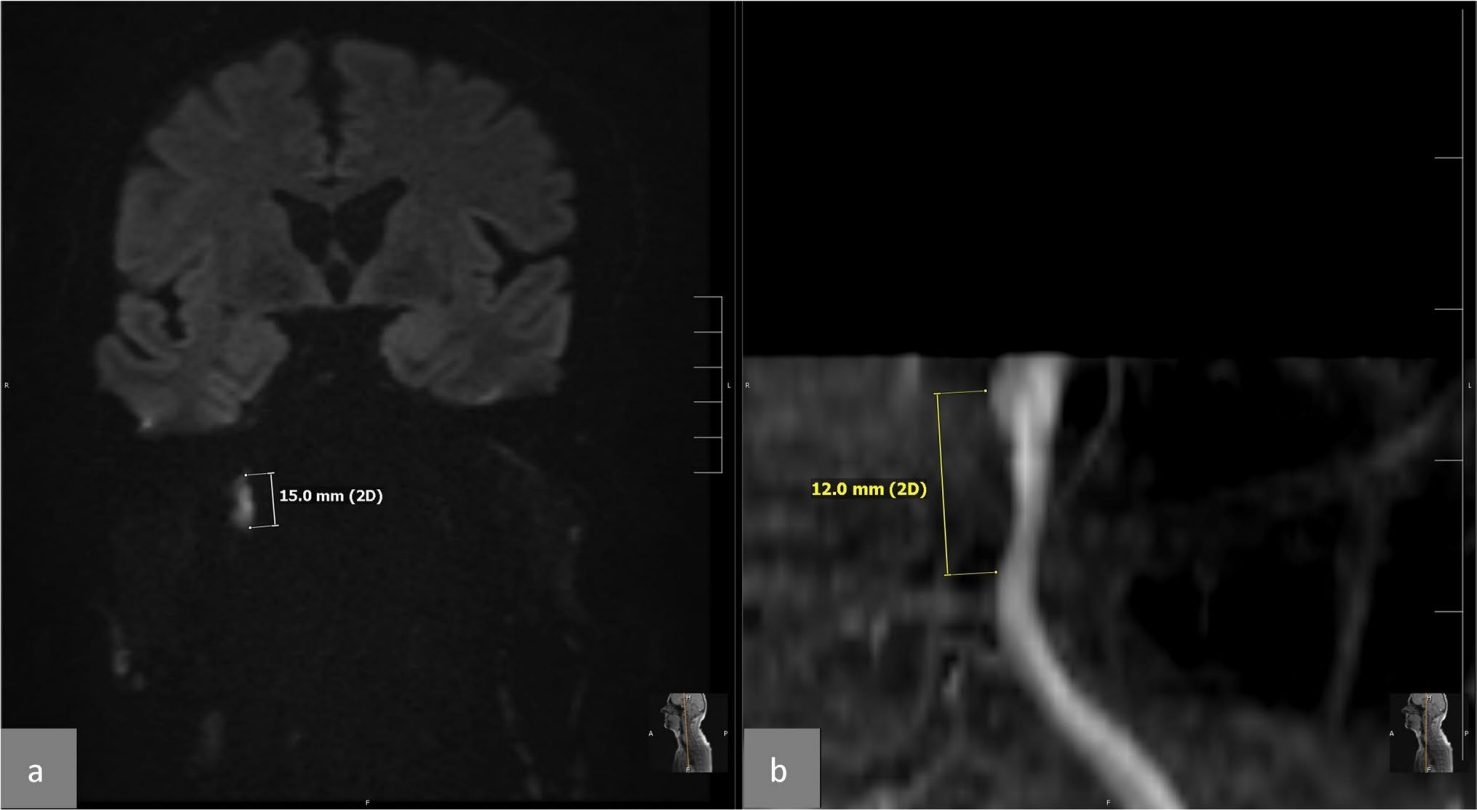
Case 10: DWI hyperintensity compared with MRA (time of flight, TOF). **a** Coronal DWI, which illustrates the hyperintensity with its length (15 mm) on the path of the right internal carotid artery (ICA) near its entry into the carotid canal in the skull base. **b** A coronal reformation of the TOF-MRA of the dissected segment of the right carotid artery with the measured length of the stenosis (12 mm). The length of the stenosis was here 3 mm shorter than on DWI

**Fig. 2:**
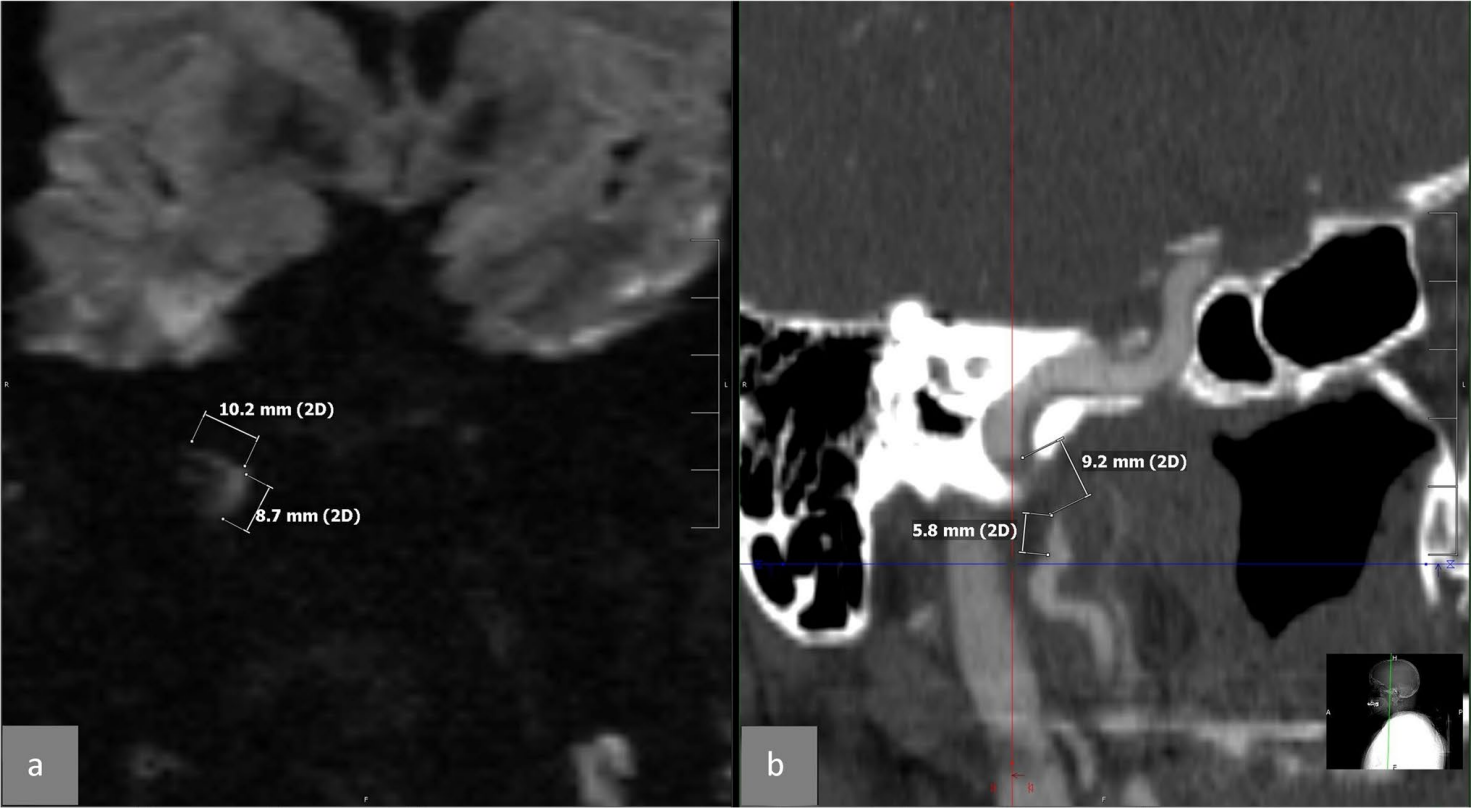
Case 19: DWI hyperintensity compared with CTA. **a** Coronal DWI, which illustrates the hyperintensity with its length (10.2 + 8.7 = 18.9 mm) on the path of the right internal carotid artery (ICA) in its entry in the carotid Chanel in the skull base. **b** A reformation of the CTA of the dissected segment of the right carotid artery with the measured length of the stenosis (9.2 + 5.8 = 15 mm). The length of the stenosis was 4 mm shorter than on DWI

**Fig. 3:**
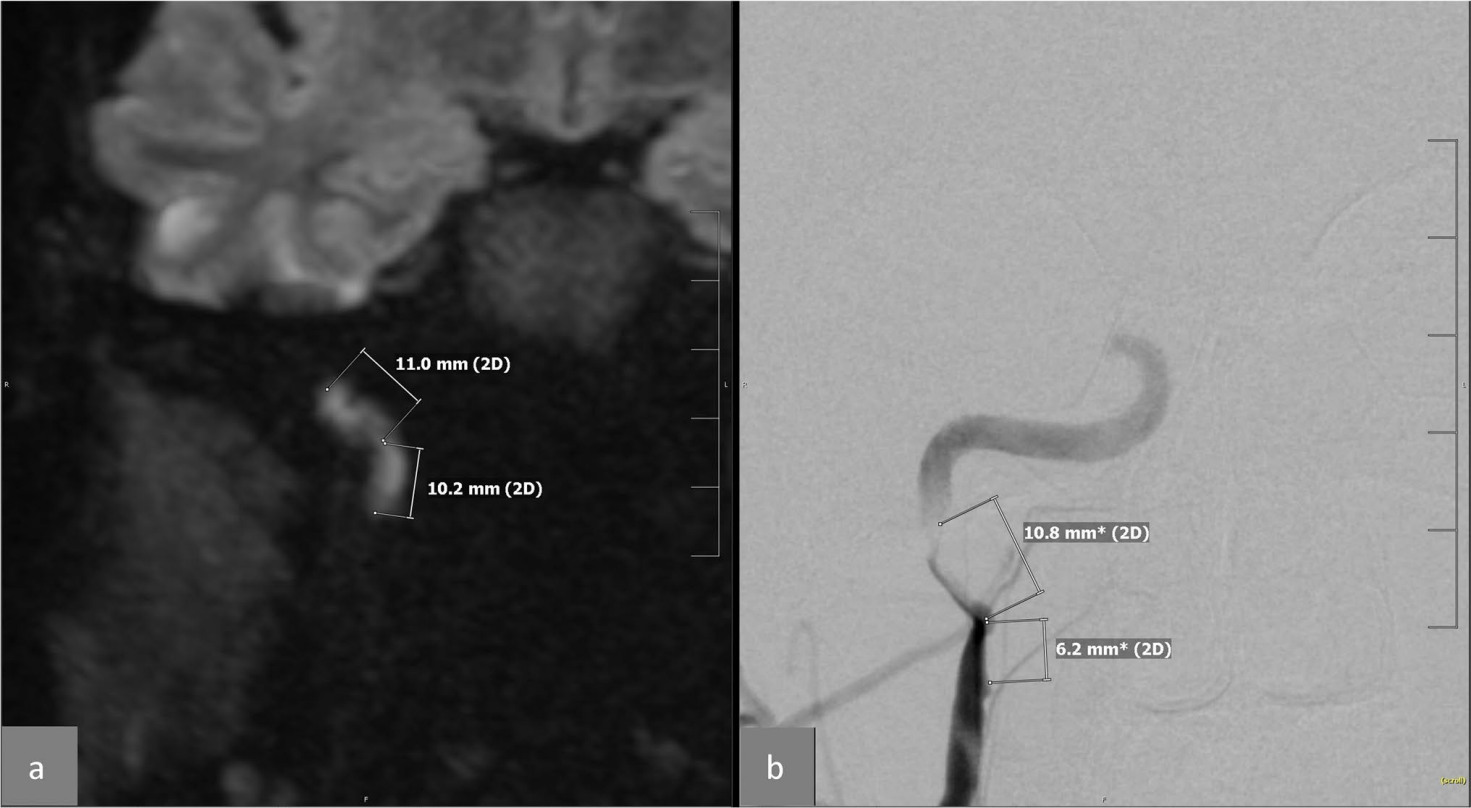
Case 27: DWI hyperintensity compared with DSA. **a** Coronal DWI, which illustrates the hyperintensity with its length (11 + 10.2 = 21.2 mm) on the path of the right internal carotid artery (ICA) near its entry into the carotid canal in the skull base. **b** A coronal DSA of the dissected segment of the right carotid artery with the measured length of the stenosis (10.8 + 6.2 = 17 mm). The length of the stenosis was 4 mm shorter than on DWI

The image analysis was performed by two neuroradiologists (MA, 3 years of experience; MD, 11 years of experience) in independent sessions. Each rater was blinded to the image analysis results of the other reader. We used a Likert-type like rating scale to grade the index vessel: grade 1 (low-grade stenosis < 30%), grade 2 (mid-grade stenosis 30% to 69%), grade 3 (high-grade stenosis ≥ 70%), and grade 4 (no lumen visible). In CTA, the absence of visible contrast agent in a cervical artery was considered to be caused by slow flow or true occlusion. The following use of the word “occlusion” describes the absence of contrast agent in the dissected artery in CTA and MRA. The length of the vessel lumen reduction (stenosis or occlusion) assumed to demonstrate the dissected area was measured on DWI images and subsequently compared to MRA, CTA, or DSA images. On DWI, we assessed the length of the signal hyperintensity in millimeter (mm) and compared this length to the length of the stenosis (in case of stenosis) and the occlusion (in case of occlusion) of the artery on MRA, CTA, or DSA images (Figs. 1, 2 and 3). In an additional session, the results of the image reading and measurements were discussed, and consensus was reached under the supervision of a third neuroradiologist (MPW, 18 years of experience) in those cases with discrepancies between both readers.

Given the fact that DSA is the accepted imaging standard for diagnosing occlusion and patency of blood vessels, we were able to determine the real length of the dissected segment of the affected artery. In the case of a “pseudo occlusion” phenomenon, we were able to determine the real length of the occlusion by moving the catheter forward and injecting contrast agent and repeating this maneuver carefully several times until we reached the dissected segment. Because this maneuver is not possible in the CTA and MRA, they are not able to determine the real dissected segment due to very slow or absent blood and contrast agent flow in the affected artery. In other words, it is not possible to differentiate between “pseudo occlusion” phenomenon and real occlusion in the CTA and MRA. That is the reason why those modalities frequently overestimate the real length of the dissected segment.

### Statistical analysis

We investigated the number of patients who developed an ischemic stroke due to CAD and the prevalence of stenosis and occlusions in CAD. We performed descriptive statistics of the data and classified our patients in 4 timing groups, < 24 h, 24–48 h, 48–72 h, and 72 h–42 d (Tables 1 and 3). In order to calculate the specificity, we selected a control group and screened for patients without CAD, some patients with and some without a stenosis or an occlusion either of the ICA or the VA, and some with and some without atherosclerotic lesions. We compared the DWI positivity in CAD patients with this control group.

**Table 1:** Study cohort with demographic and imaging findings

CASE NUMBER, SEX, AGE	DWI HYPERINTENSITY AND TIME TO DWI ASSESSMENT	IMH, LENGTH ON DWI IN MM	TYPE OF THE USED ANGIOGRAPHY	STENOSIS, OCCLUSION, OR ANEURYSM	LENGTH IN THE ANGIOGRAPHY IN MM	THE DIFFERENCE OF THE LENGTH BETWEEN DWI AND ANGIOGRAPHY	WHICH ONE IS LONGER, THE USED ANGIOGRAPHIC MODALITY OR DWI
CASE 1 f, 39 Y	Positive 24–48 h	33	MRA	Occlusion	50	17	Angiography
CASE 2 m, 39 y	Positive 12 d	11	MRA	Aneurysm	11	0	Equal
CASE 3 m, 68 y	Negative 21 h	No DWI hyperintensity	MRA	Occlusion	*Not measurable	*No DWI hyperintensity	*Not measurable
CASE 4 f, 54 y	Positive 24–48 h	35	MRA	Low-grade stenosis	42	7	Angiography
CASE 5 f, 36 y	Positive 13 d	26	MRA	Occlusion	60	34	Angiography
CASE 6 m, 38 y	Positive 48–72 h	21	MRA	Low-grade stenosis	18	3	DWI
CASE 7 m, 29 y	Positive 18 d	17	MRA	Mid-grade stenosis	8	9	DWI
CASE 8 m, 51 y	Positive < 24 h	28	MRA	Low-grade stenosis	27	1	DWI
CASE 9 m, 49 y	Positive 11 d	27	MRA	High-grade stenosis	22	5	DWI
CASE 10 m, 65 y	Positive 42 d	20	MRA	Mid-grade stenosis	45	25	DWI
CASE 11 m, 22 y	Positive 1.5 h	18	MRA	Low-grade stenosis	19	1	Angiography
CASE 12 m, 47 y	Positive 24–48 h	23	CTA	Occlusion	30	7	Angiography
CASE 13 m, 53 y	Positive 24–48 h	63	CTA	High-grade stenosis	65	2	Angiography
CASE 14 m, 55 y	Positive 4 d	25	CTA	High-grade stenosis	43	18	Angiography
CASE 15 m, 55 y	Positive 4 d	26	CTA	High-grade stenosis	29	3	Angiography
CASE 16 M, 45 y	Positive 20.5 h	60	CTA	Occlusion	*Not measurable	*Not measurable	*Not measurable
CASE 17 f, 17 y	Positive 19 h	15	CTA	High-grade stenosis	18	3	Angiography
CASE 18 F, 37 y	Positive 9 h	20	CTA	Occlusion	70	50	Angiography
CASE 19 m, 54 y	Positive 3 h	19	CTA	Occlusion	15	4	DWI
CASE 20 m, 24 y	Positive 21.5 h	30	CTA	Occlusion	110	80	Angiography
CASE 21 f, 53 y	Positive 2 h	27	DSA	Occlusion	39	12	Angiography
CASE 22 F, 37 y	Positive 4 d	15	DSA	Occlusion	14	1	DWI
CASE 23 m, 60 y	Positive 24–48 h	27	DSA	Occlusion	28	1	Angiography
CASE 24 m, 47 Y	Positive 24–48 h	39	DSA	Occlusion	47	8	Angiography
CASE 25 m, 54 y	Positive 24–48 h	41	DSA	High-grade stenosis	45	4	Angiography
CASE 26 m, 54 y	Positive 17 h	27	DSA	Occlusion	25	2	DWI
CASE 27 m, 49 y	Positive 5.5 h	21	DSA	Occlusion	17	4	DWI
CASE 28 F, 72 y	Positive 26 d	48	DSA	Occlusion	50	2	Angiography
CASE 29 m, 49 y	Negative 4.5 h	No DWI hyperintensity	DSA	Occlusion	80	No DWI hyperintensity	Not measurable
CASE 30 m, 59 y	Positive 19 d	20	DSA	Occlusion	22	2	Angiography

The first column shows the case number. The second column shows whether the dissection is hyperintense on the DWI-sequence or not (DWI positive means that there was a DWI hyperintensity and DWI negative means that there was no DWI hyperintensity). The third column shows the length of the DWI hyperintensity (which represents the length of the length of the IMH). The fourth column shows the type of the used angiography (to which the DWI length was compared). The fifth column shows the grade of the stenosis (or occlusion) of the affected artery. The sixth column shows the length of the stenosis or occlusion of the artery. The seventh column shows the difference of the length between DWI and angiography, and the eighth column shows which one is greater, the length on DWI or on angiography

In order to evaluate the accuracy of assessment of the dissection length, we calculated the absolute difference between the length of the DWI hyperintensity and the length of the dissection in MRA, CTA, and DSA (Table 1). After that, we calculated the mean deviation (MD) and standard deviation (SD) for each angiographic modality separately (Table 2).

**Table 2:** Modified four-field table showing DWI positivity as a function of the time interval between the onset of CAD and the time of MRI

*MRI time after onset*	< *24 h* n = *12*	*24–48 h* n = *7*	*48–72 h* n = *1*	*72 h–42 days* n = *10*
*DWI positive*	83%	100%	100%	100%
*DWI negative*	17%	0%	0%	0%

## Results

### Patient cohort

We included 28 patients (8 females and 20 males, age 17–78 years) presenting with in total 30 confirmed CAD of one or more of the carotid or the vertebral arteries. Two of these patients had a bilateral dissection of the internal carotid arteries resulting in a total number of 30 dissected arteries. No imaging findings suggestive of arteriosclerosis were observed on the area of the dissection. Demographic and clinical information regarding the study subjects are given in Table 1. There was an effect of sex: It could be noticed that males (*n* = 22, sixteen of them had ICA dissection and six had VA-dissection) are more often affected with dissection of ICA and VA than females (*n* = 8, six of them had ICA dissection and two had AV dissection).

### CAD manifestation pattern

The ICA (80%) was more often affected than the VA (20%). The right side was more often affected than the left side. In the ICA group, the right side involved 13 patients, and the left side was affected in 11 patients. Four patients had VA dissection on the right side compared to two patients with a left sited VA dissection.

### Ischemic stroke in the brain

In all of VA dissections (*n* = 6 of 6 patients, 100%), the patients had a brain infarction documented on MRI or CT of the brain. In fifteen patients of twenty-two patients of ICA dissections (*n* = 15 of 22 patients, 68%), the patients had a brain infarction documented on MRI or CT of the brain.

### Stenosis, occlusion, and pseudo aneurysm after dissection

The arterial dissection in our patient population resulted either in a stenosis, occlusion, or (pseudo)aneurysm formation. More than a half of the patients (56%) had an occlusion, four patients (13%) had a low-grade stenosis, two patients (7%) had a mid-grade stenosis, six patients (20%) had a high-grade stenosis, and 1 patient (3%) had a (pseudo)aneurysm.

In addition, 20 control subjects without CAD vessel lesions were included (8 females and 12 males, age 24–89 years). Ten patients presented with a stenosis of the ICA, two with low-grade, two with mid-grade, and six with high-grade stenosis (6 presenting with atherosclerotic lesions and 4 without atherosclerotic lesions); five patients with an occlusion of the ICA (two patients presenting with atherosclerotic lesions and three patients without atherosclerotic lesions); two patients presenting with a stenosis of the VA, 1 mid-grade and one patient with high grade stenosis (one patient presenting with an atherosclerotic lesion and one patient without an atherosclerotic lesion); and three patients presenting with an occlusion of the VA (two patients presenting with atherosclerotic lesions and one patients without an atherosclerotic lesion).

### Diagnostic performance of DWI

The results of all patients regarding IMH length on DWI, confirmatory image modality (MRA, CTA, DSA), vessel involvement (stenosis, occlusion, aneurysm), and length of the CAD manifestation are presented for each individual patient in Table 1. A signal hyperintensity on DWI suggestive of CAD was detected in 28 of the 30 dissected vessels resulting in a sensitivity of 93%. There were no DWI hyperintensities detectable in the control group patients resulting in a specificity of 100%.

**Fig. 4:**
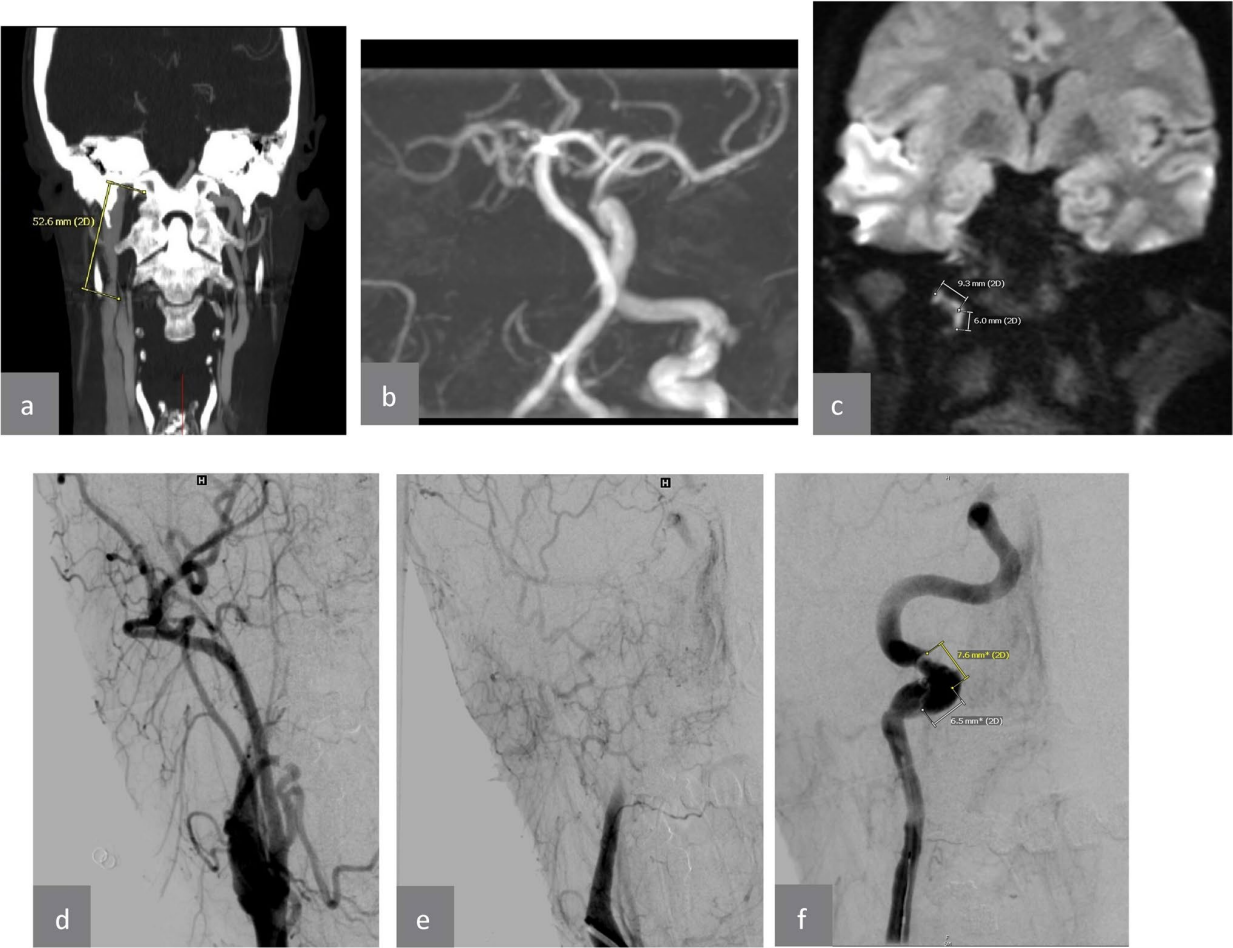
A case with DSA, CTA, MRA and DWI that shows that CTA and MRA were misleading and that DWI matched DSA. **a** A coronal CTA shows a contrast agent stop immediately distal the ICA origin that supposes a long distance occlusion. **b** A coronal TOF reconstruction shows a long distance absent flow signal in the right ICA, which supposes also a long distance occlusion. **c** A coronal DWI in contrary to the previous modalities shows a short distance hyperintensity in the ICA wall at the skull base. **d** A coronal DSA shows a contrast agent stop immediately distal the ICA origin, while the catheter tip was still located in the common carotid artery (CCA). **e** A coronal DSA shows contrast agent more distal and in a longer segment of the ICA after the catheter tip was placed in the ICA origin. **f** A coronal DSA after replacing the catheter and moving it carefully until there was a resistance against forward catheter pushing that shows now the real dissected segment of the ICA, which matches the length of DWI hyperintensity

Regarding the time interval between symptom onset and MRI, we subdivided the CAD cases into four different groups (Table 2). In the group presenting within 24 h after symptom onset, we detected a hyperintense signal intensity in the affected vessel in 10 of 12 cases resulting in a sensitivity of 83%. In all patients of the groups presenting to the MRI between 24–48 h, 48–72 h, and 72 h–42 days after symptom onset, we identified a hyperintense signal intensity in the affected vessel (100% sensitivity). The high signal intensity on the DWI was even present in one participant within the first 1.5 h (Table 1, case number 11, Figs. 1, 2, 3 and 4).

### Length of vessel involvement and DWI hyperintensity

The MD in 27 measurable cases was 11.6 mm, and the standard deviation SD was 17.9 mm. The three highest outliers are 80 mm (case 20, CTA), 50 mm (case 18, CTA), and 34 mm (case 5, MRA). These considerable variations were not present in those cases who underwent DSA, which indicates that the CTA and MRA could overestimate the real length of the dissected segment of the artery (detailed results in Table 3).

**Table 3:** The MD and SD (in mm) of the DWI hyperintensity length, the lesion length (occlusion, stenosis or aneurysm in each angiographic modality), and the difference between the DWI hyperintensity length and the lesion length in each angiographic modality

	*DWI hyperintensity*		*Lesion length*		*Length difference between DWI hyperintensity and lesion length in the following modalities*	
*Modality*	MD	SD	MD	SD	MD	SD
MRA	6.2	7.5	15.3	17.8	9.1	11.5
CTA	9.4	15	25.6	32.3	22.1	28.9
DSA	8.8	10.9	11.8	13.6	2.7	3.7

The results are separated by all three groups (MRA, CTA, and DSA). All patients in each group underwent one of the mentioned angiographic examinations and an MRI including DWI. The first column “Modality” shows which angiographic modality (MRA, CTA, or DSA) was used in addition to the DWI and compared with the DWI findings. The second column “DWI hyperintensity” shows the MD and SD of the length of DWI hyperintensity in each group separately. The third column “lesion length” shows the MD and SD of the lesion length (occlusion, stenosis, or aneurysm) in each group separately. The fourth column shows the MD and SD of length difference between DWI hyperintensity and lesion length in each group separately

For the calculation of the MD and SD in those cases, in which the real length of the dissection was precisely measured, we considered the cases with available DSA separately. In these cases, the MD was 2.7 mm, and SD was 3.7 mm. For the calculation of the MD and SD in patients with a stenosis and no apparent vessel occlusion, we excluded the patients presenting with an apparent occlusion. In this sub-analysis, the MD was 5.3 mm, and SD was 7.4 mm. Figures 1, 2 and 3 demonstrate examples regarding the assessment of the length of the stenosis.

## Discussion

The main objective of this study was to investigate the role of the diffusion weighted imaging (DWI) in detecting the acute dissection of ICA and VA as well as the assessment of the length of the dissected vessel. In our patient cohort, we found that DWI is highly sensitive for the detection of dissected cervical arteries even in the first hours after symptom onset and can reliably assess the length of the dissection.

Despite advances in imaging techniques regarding the assessment of small and medium-size arteries including vessel wall imaging, an early and accurate diagnosis of CAD remains challenging in the clinical routine setting [6]. The most serious consequence of CAD, in particular when the diagnosis is missed or delayed, are brain infarctions. The prevalence of ischemic stroke after ICA and VA dissection has been reported to be 60% relatively high particularly in VA dissections [12, 13]. In our study, brain infarctions were present in 100% of the patients presenting with VA dissections and in 68% of the patients presenting with ICA dissections. The different prevalence if strokes in our study population might be due to inclusion bias.

Preliminary studies including a limited number of patients suggested first evidence that DWI-MRI might be a sensitive method in the detection of IMH suggestive of CAD [9–11]. In fact, the results of our study confirm this observation showing a sensitivity in terms of dissection diagnosis of 83% in the first 1.5–24 h and of 100% after 24 h. Overall, the sensitivity was 93%. These results suggest that DWI-MRI is a considerable alternative of the fat-suppressed T1w and T2w sequence or at least a diagnostic tool in addition to these pulse sequences in order to increase the diagnostic accuracy in the first 7 days, particularly within the first 48 h after symptom onset.

In addition to the testing of the diagnostic performance of DWI-MRI in acute CAD, our data demonstrates the potential to estimate the real length of the dissected segment in a fast and noninvasive way and without the need of an angiographic examination. Our data suggest that DWI-MRI can be used to assess the length of the dissection in a non-invasion way and even before starting an angiographic attempt of recanalization. According to our clinical experience, this is highly relevant to select the right length of the stent when we try to perform a mechanical recanalization of the dissected artery. The MD was 11.6 mm, and the SD was 17.9 mm. In DSA cases, the MD was 2.7 mm, and SD was 3.7 mm, and if there was just a stenosis of the artery without an apparent occlusion, the MD was 5.3 mm, and SD was 7.4 mm. These results are more than acceptable and very helpful for the decision-making and planning of interventional recanalization procedures.

As above mentioned, using MRA or CTA, it is not possible to reliably differentiate between real and “pseudo-occlusions” the real length of CAD that could be extremely overestimated [14]. This issue is stressed by three extreme results of the occlusion length. For example, the case number 22 showed a long distance apparent occlusion in the CTA and MRA, but in the DSA a short distance dissection, which exactly matches the DWI hyperintensity (Fig. 4). We suppose that the apparent occlusion in the above-mentioned cases with extreme differences between non-dynamic imaging and DSA is most likely over estimated (“pseudo occlusion” phenomenon), which does not represent the real dissection length, the IMH, and its length. Patients with ICA and VA dissections develop very often vessel occlusions (56%) followed by high-grade stenosis (20%), whereas pseudo aneurysms are rare after CAD (3%).

One considerable limitation of our study design is the retrospective analysis and the lack of standardization of the image acquisition protocols. In addition, histopathology data as a diagnostic gold standard in order to definitely confirm the clinical and imaging diagnosis of CAD is missing. However, this is also the case in the vast majority of CAD cases in clinical practice considering clinical and imaging data as sufficient to diagnose CAD.

Another limitation of our study is the absence of the direct comparison to the sensitivity and specificity of the T1- und T2-weighted sequences due to the absence of such statistics in the included previous studies, which just described the limitation of T1- und T2-weighted sequences in the early course of CAD. In a prospective study could this point be covered by planning these sequences regularly.

It is remarkable that this high sensitivity and specificity of DWI-MRI is even present despite this limitation. It can be anticipated that the potential diagnostic value of DWI-MRI in the detection of early CAD can be further improved in the setting of strictly standardized MR image acquisition using one single MR system with a scanner optimized image acquisition protocol.

In conclusion, our study provides further evidence that DWI-MRI might be a sensitive method in the detection of IMH suggestive of CAD. Our data and clinical experience suggest that DWI-MRI can represent a valuable diagnostic tool in addition to fat-suppressed T1w and T2w sequences in order to increase the diagnostic accuracy with regard to a diagnosis of CAD in the early disease course. Further multi-center prospective studies applying standardized image acquisition are needed to finally support the use of DWI-MRI for diagnosis of CAD and determination of length of IMH in the clinical routine setting and to investigate the time interval in which dissected arteries show a high signal intensity on DWI.

## Data Availability

The data was available for all authors.

## Abbreviations

AD: Arterial dissection
CAD: Cervical artery dissection
CTA: Computed tomography angiography
DSA: Digital subtraction angiography
DWI: Diffusion weighted imaging
FLAIR: Fluid-attenuated inversion recovery
ICA: Internal carotid artery
CCA: Common carotid artery
IPH: Intraplaque hemorrhage
IMH: Intramural hematoma
MD: Mean deviation
MRA: Magnetic resonance angiography
MRI: Magnetic resonance imaging
PTA: Percutaneous transluminal angioplasty
SD: Standard deviation
ST: Slice thickness
T: Tesla
TE: Echo time
TR: Repetition time
TOF: Time of flight
VA: Vertebral artery

## Code availability (software application or custom code)

Does not apply.
